# Decreased Functional Connectivity in the Reward Network and Its Relationship With Negative Emotional Experience After Total Sleep Deprivation

**DOI:** 10.3389/fneur.2021.641810

**Published:** 2021-05-12

**Authors:** Ying Zhang, Cimin Dai, Yongcong Shao, Jiaxi Peng, Yan Yang, Yanhong Hou

**Affiliations:** ^1^Department of Psychology Medical, The Eighth Medical Center, Chinese PLA General Hospital, Beijing, China; ^2^Department of Stress Disorder Treatment, The Eighth Medical Center, Chinese PLA General Hospital, Beijing, China; ^3^School of Psychology, Beijing Sport University, Beijing, China; ^4^Department of Radiology, The Eighth Medical Center, Chinese PLA General Hospital, Beijing, China

**Keywords:** sleep deprivation, functional connectivity, nucleus accumbens, reword network, fMRI

## Abstract

Sleep deprivation (SD) induces a negative emotional experience due to a prolonged time spent awake. However, few studies have focused on the mechanism underlying communication within brain networks or alterations during this emotional deterioration. We propose that negative reward judgment is important in poor emotional processing after SD, which will be reflected in functional connectivity in the reward network. We sought to analyze alterations in functional connectivity within the reward network and cerebral cortex. Furthermore, we analyzed changes in functional connectivity correlation with negative emotional experience after SD. Twenty-six healthy volunteers participated in this study. Two resting-state fMRI scans were obtained from the participants, once during resting wakefulness, and once after 36 h of total SD. The bilateral nucleus accumbens (NAc) was selected as a seed region for region of interest (ROI)-to-ROI functional connectivity analysis. Correlation analyses between functional connectivity alterations within the reward network and negative emotional experience were also performed. We found that SD decreased functional connectivity between the left NAc and anterior cingulate cortex (ACC) compared with resting wakefulness. There was a decreased functional connectivity with the ACC and right inferior frontal gyrus (IFG) after SD in the right NAc. Furthermore, decreased functional connectivity between the right NAc and right IFG, and NAc and ACC was negatively correlated with emotional experience scores. Sleep deprivation decreased functional connectivity within the reward network. This may be associated with the enhanced negative emotional experience that was found after total sleep deprivation.

## Introduction

Sleep deprivation (SD) can cause serious harm to humans. Beyond its negative impact on cognitive brain function, such as attention, working memory (1), and decision making (2), SD also results in deterioration of emotional experience (3, 4). Studies have shown that SD leads to poor mood (5) and increases nerve sensitivity to negative emotional stimuli (6). For example, extending waking time induces irritability, aggressiveness, and increased anxiety (7). Furthermore, reduced control over emotional states can lead to more harmful actions (8). Emotional instability can negatively impact cognitive function and induce serious human failure, which can result in labor accidents (3, 9).

Deterioration in emotional states following SD is associated with the emotional processing network in the brain (10). In a study using negative emotional images, participants report a more negative emotional experience after 24 h of total SD (11). Killgore et al. (3) have proposed that emotional instability was positively correlated with increased amygdala activity following SD. Moreover, functional connectivity between the amygdala and cerebral cortex is altered following SD (12). Furthermore, functional connectivity between emotional networks is positively correlated to Profile of Mood States (POMS) questionnaire scores, which can detect deteriorations in emotional state (8). Positron emission tomography studies have shown enhanced activity in the limbic system, which is associated with emotion, following SD (13). In addition, several studies have indicated that an increased emotional state after SD is correlated with the emotional control of brain functioning (10, 14). Together, these studies confirm that SD induces a decline in emotional state and emotional instability as SD is prolonged.

SD can impact on emotional decision making (15). Emotions can be adjusted by reward or punishment; therefore, decision making associated with loss and gain can be influenced by emotional state following SD. Several studies have been found changes in risk-taking behavior following SD (16–18), leading to more dangerous or risky decisions. Insufficient sleep enhances the sensitivity of reward system (19, 20). Bad emotional experiences are linked to activation of the reward network, such as the nucleus accumbens (NAc), anterior cingulate cortex (ACC), and orbitofrontal cortex (OFC), which have been studied in relation to substance addiction (21). In contrast to SD, new evidence suggests that the existence of sleep is beneficial in supporting several types of emotional reward brain function (22).

However, few studies have focused on reward network communication and emotional instability after SD. Furthermore, changes to the reward network, and the relationship between the reward network and negative emotional experiences have not been fully understood.

Recently, resting-state fMRI has been employed to investigate various brain functions. Resting-state functional connectivity is a useful tool to develop information regarding the communication between and within brain networks (23, 24). In this study, we investigated the changes in functional communication before and after SD. In addition, we assessed the relationship between functional connectivity changes within the reward network and negative emotional experiences. We hypothesized that (1) SD would induce a decline in functional connectivity in the reward network; and (2) this is positively correlated with negative emotional experiences. We performed resting-state fMRI scans before and after 36 h of total SD. The bilateral NAc was selected as the seed region for functional connectivity analysis.

## Methods

### Participants

The data analyzed in this paper consisted of two parts. The first part of the data came from the previous experiment (12), including fourteen subjects from Beijing Normal University. The second part of the data were conducted in 2018, with nineteen participants from Beihang University. According to the completeness of fMRI data and behavioral data, incomplete ones (six without behavioral data and one without fMRI data) were removed. Finally, there were eight left in the first part and eighteen left in the second part. All the twenty-six subjects were healthy male students from University participated in the study as paid participants and were selected according to the same criteria. Participants were right-handed, with normal or corrected-to-normal vision, and normal cognitive function and intelligence (Raven IQ test >100). Exclusion criteria were: peripheral or central nervous system disease; cardiovascular disease and/or hypertension; cataracts and/or glaucoma; pulmonary difficulties; audiological difficulties; or substance abuse (including alcohol). Participants were asked to abstain from tobacco smoking, drink <1/2 cups (150 ml) coffee or tea per day, and have a regular sleep pattern with at least 8 h of sleep for at least 1 week prior to the experiment. All participants fully understood the protocol and provided informed consent before the experiment began. The experiment was approved by the Ethics Committee of The General Hospital of PLA (Beijing, China).

### Experimental Paradigm

**Figure 1:**
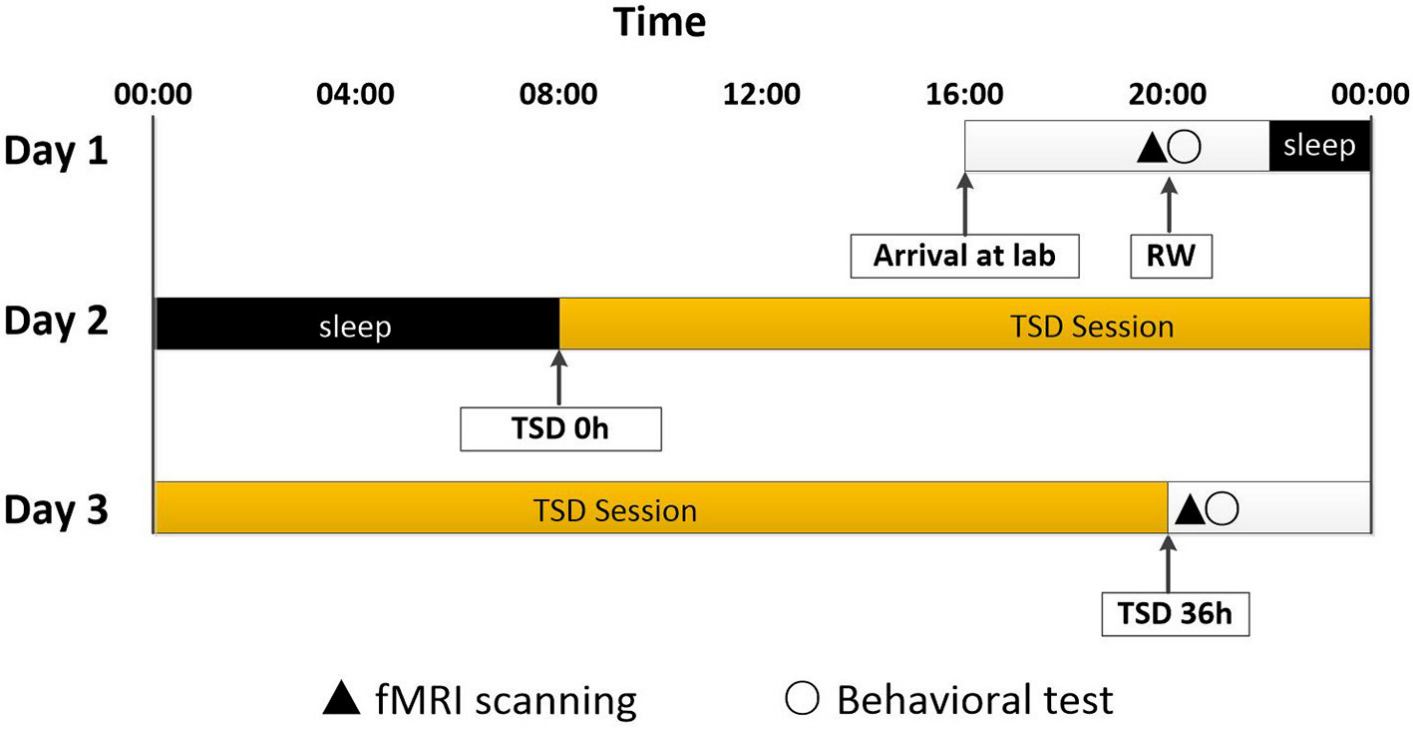
Experimental design and protocol. TSD began after a routine nocturnal sleep period, at 08:00 on Day 2, and ended at 20:00 at Day 3. Participants were required to stay awake for 36 h during the whole TSD session. Participants performed fMRI scanning and behavioral tests at 20:00 on Day 1. After 36 h TSD, fMRI scanning and behavioral tests was performed at approximately 20:00 on Day 3 for all participants. TSD, total sleep deprivation; RW, rested wakefulness.

All participants completed the experiment according to the unified standard process. They arrived at the laboratory at 16:00 on the first day of the experiment. They completed a demographic questionnaire and other scales, including the self-rating anxiety scale (SAS), self-rating depression scale (SDS), and Pittsburgh sleep quality index (PSQI). The rested wakefulness (RW) session of fMRI scanning was conduct at 20:00 on Day 1. Following a routine nocturnal sleep period, total sleep deprivation (TSD) started at 08:00 on Day 2 until 20:00 on Day 3. Participants were required to stay awake for 36 h during the whole TSD session. Participants performed the TSD fMRI scan at 20:00 on Day 3. After each scan session, the visual analog scale (VAS) and POMS questionnaires were used, which measured the subjects' alertness and emotional state, respectively. An experimental design schematic is shown in Figure 1.

The experiment was performed with paramedics present throughout the process. During continuous behavioral monitoring, each participant was accompanied by a partner to help them stay awake at night. Participants were not permitted to leave the lab during the experiment, except when they were escorted to the fMRI equipment for scanning.

### Data Acquisition

Eight subjects' data have been previously studied (12). The rest of the data were obtained with following process.

Participants were positioned in the scanner with their heads comfortably restrained by foam padding to reduce head movement. Earplugs were used to reduce the noise of the scanner. During the resting-state scans, participants were instructed to keep their eyes closed, remain as motionless as possible, and clear their head of any particular thoughts. A pulse oximeter was attached to the participant's finger to record cardiac activity. In addition, participants wore a pressure belt around the abdomen to record respiratory activity. Cardiac and respiratory signals were synchronized with fMRI data to ensure that physiological changes were removed during the regression analysis.

Standard static functional images and T2^*^-weighted echo plane images were obtained using a Siemens 3.0T scanner with a birdcage RF head coil. Scanning sessions included: (i) localization, (ii) T1-FLAIR (RT: 8.208 ms, RE: 3.22 ms, FoV = 256 mm × 256 mm, Sag slice number = 192, voxel size = 1 × 1 × 1 mm^3^, no slice gap), and one (iii) resting-state scan. A single EPI sequence (TR: 2 s, TE: 30 ms, Flip Angle: 90°, FoV: 256 mm × 256 mm, 3.75 × 3.75 mm planar resolution) covering 35 oblique slices across the whole brain was used to measure the blood oxygenation level-dependent (BOLD) signal. There were 190 volumes in total. Researchers monitored the subjects *via* camera and reminded them to stay awake using a microphone before each scan. After each part of the session, participants were asked if they were awake during the previous trial, and all confirmed that they had been awake.

### Data Pre-processing

SPM 12 (University College London, available at http://www.fil.ion.ucl.ac.uk/spm/) and CONN toolbox v17a (Neuroimaging Informatics Tools and Resources Clearinghouse, available at https://www.nitrc.org/projects/conn) were employed for functional image preprocessing (25). The first 10 volumes of the functional time series of each epoch were discarded for signal stabilization and to accustom participants to scanning noise. First, registration of the first volume was performed to correct for head movement. Rotation and movement of all participants was within 2 mm or 2° in the x, y, and z plane. Volumes were normalized to the standard EPI template in Montreal Neurological Institute space, and restored to 3 × 3 × 3 mm^3^. The resulting images were spatially smoothed with a 6-mm full width at half-maximum Gaussian kernel. Subsequently, the CompCor function was used for spatial and temporal preprocessing to minimize the impact of motion and physiological noise factors, and define and remove confounds in the BOLD signal (26). Regression of first order derivative terms for the whole brain, ventricular, and white matter signals were also included in the correlation preprocessing. This regression process was used to reduce the influence of spurious variance on neuronal activity.

### Functional Connectivity Analysis

CONN is a MATLAB-based cross-platform software for computing and analyzing functional connections in brain regions based on fMRI signals (25). This software has been widely used and its reliability has been well-documented. We scanned the region of interest (ROI)-to-ROI connectivity matrix, tested hypotheses, and visualized data using the CONN toolbox implemented in Matlab v. 2016 and SPM12 (25). ROIs (*n* = 132) of the whole brain were drawn from the template provide by CONN (conn/rois/atlas.nii), which included the bilateral NAc (27). All ROIs were imported into the CONN Toolbox.

Functional connectivity measures were computed between seed areas for ROI-to-ROI analysis and to identify patterns of ROI-to-ROI connectivity. The CONN toolbox was used to obtain a linear measure of functional connectivity based on bivariate correlation, and bivariate regression coefficients and their associated multivariate measures of semi-partial correlation and multivariate regression coefficients (25). We choose to use bivariate correlation to get functional connections. We compared functional connectivity between the RW and TSD scans using two-tailed paired *t*-tests. The resulting statistical maps were set with *p* < 0.05 at the cluster level (FDR corrected). For maps that did not meet this criterion, a relaxed threshold of *p* < 0.001 at the cluster level was also used.

### Behavioral Correlations

To investigate the relationship between changes in functional connectivity and mood before and after SD, we conducted a correlation analysis. We calculated the change of distress sore (based on participants' responses on the POMS questionnaire) before and after SD and the pearson correlation coefficient between it and the degree of functional connectivity reduction. Data were deemed to be statistically significant if p_FDR−corrected_ < 0.05 (Benjamini and Hochberg FDR).

## Results

### Physiological Data

Demographic data, psychological traits, and sleep characteristics are shown in Table 1. The mean respiration and heart rate before and after TSD was compared using paired *t*-tests. There were no differences in the heart or respiratory rate between the RW and TSD conditions (*p* > 0.05, Table 1). Paired *t*-tests revealed significant differences between the two conditions in alertness (*p* < 0.001), Anger-Hostility (AH; *p* < 0.001), Fatigue-Inertia (FI; *p* = 0.003), the total score of the POMS (TMD; *p* = 0.039), and distress scores (*p* < 0.001; Table 1).

**Table 1:** Demographic data, psychological traits, and sleep evaluation (*n* = 29).

	**RW state**	**TSD state**	***t***	***p***
Age (years)	23.62 ± 3.21	-	-	-
Male [n (%)]	29 (100%)	-	-	-
BMI (kg/m^2^)	22.38 ± 2.10	-	-	-
Education (years)	15.85 ± 1.52	-	-	-
SAS	23.42 ± 15.29	-	-	-
SDS	23.92 ± 14.12	-	-	-
PSQI	3.31 ± 1.16			
VAS (Alertness)^a^	7.96 ± 1.22	5.54 ± 2.14	6.572	<0.001
POMS^b^				
TA	25.04 ± 5.24	25.73 ± 3.34	−0.631	0.534
DD	39.85 ± 7.46	38.42 ± 6.64	0.957	0.348
AH	28.27 ± 5.10	32.62 ± 5.76	−7.722	<0.001
VA	28.50 ± 7.11	29.19 ± 5.66	−0.354	0.726
FI	18.62 ± 2.48	20.77 ± 3.54	−3.251	0.003
CB	21.81 ± 4.09	22.04 ± 3.64	−0.251	0.804
TDM	162.08 ± 19.80	168.77 ± 16.61	−2.177	0.039
Distress^c^	1.77 ± 0.82	4.35 ± 0.49	−14.565	<0.001

*BMI, body mass index; SAS, self-rating anxiety scale; SDS, self-rating depression scale; PSQI, Pittsburgh sleep quality index; POMS, profile of mood states;*

a *Alertness, based on participants' response to on the visual analog scale (“alertness”);*

b *The POMS scale consists of six subscales. The POMS consists of six subscales: TA, tension-anxiety; DD, depression-dejection; AH, anger-hostility; FI, fatigue-inertia; CB, confusion-bewilderment; VA, vigor-activity. TMD, the sum of this six components;*

c *Distress based on participants' responses to AH subscale of the POMS questionnaire (“distress”)*.

### Functional Connectivity of the NAc

**Figure 2:**
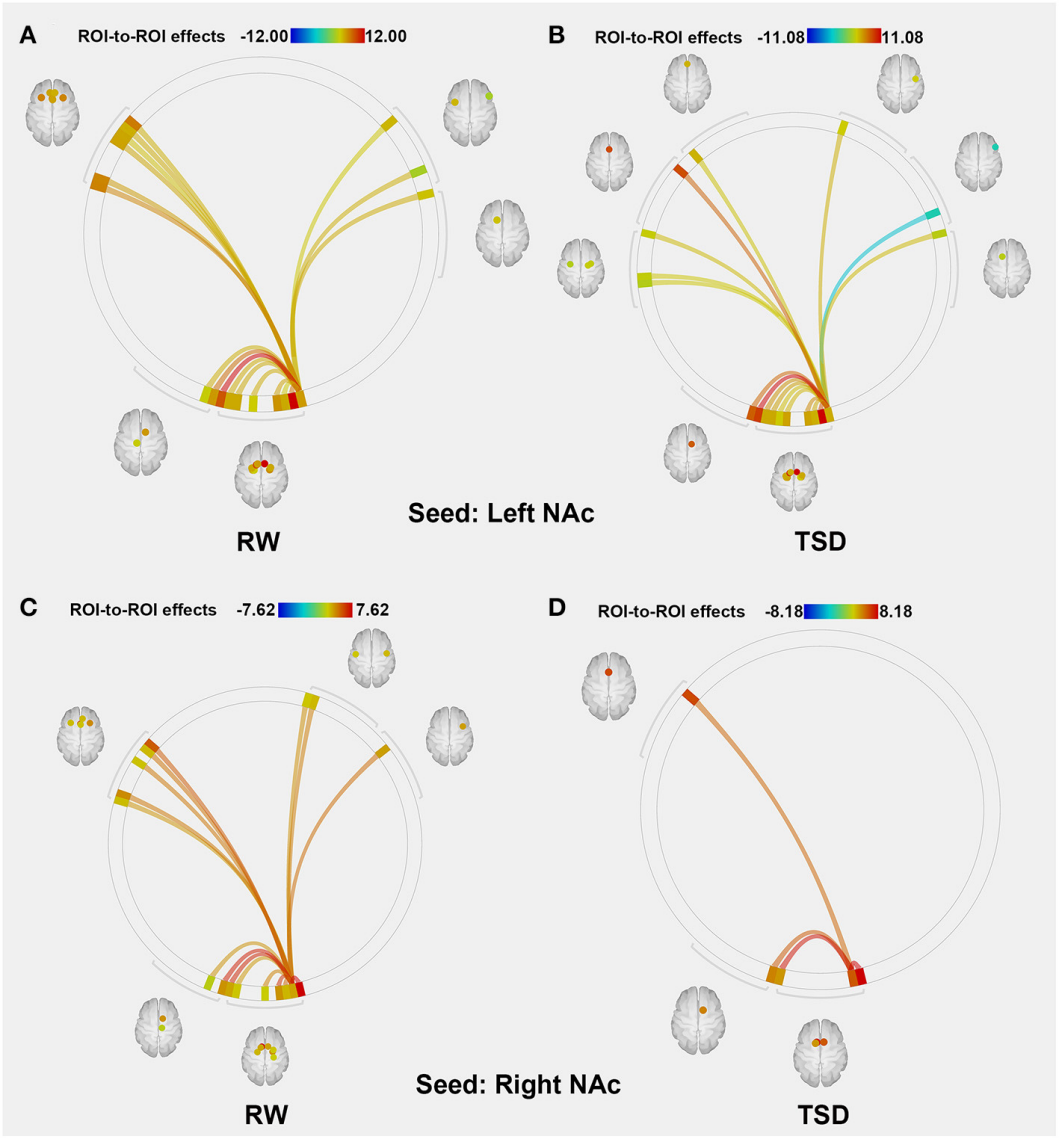
ROI-to-ROI connectivity of NAc regions at RW and following TSD. **(A,B)** RW and TSD scans showing connectivity of the left NAc. **(C,D)** RW and TSD scans showing connectivity of the right NAc, *p* < 0.05, FDR set-wise corrected for all comparisons across the entire network. ROI, region of interest; NAc, nucleus accumbens; TSD, total sleep deprivation; RW, rested wakefulness.

The purpose of this part of the analysis was to find out the functional connectivity between the left/right NAc and other nodes of reward network at RW or SD. Details were shown in Tables 2, 3 and Figure 2.

**Table 2:** ROI-to-ROI functional connectivity statistics of the NAc at RW.

**Target region**	**MNI center**	***t***	**Uncorrected**	**FDR-corrected**
			***p*-value**	***p*-value**
Left accumbens	(−9,11,−7)			
Left caudate	(−13,9,10)	12.00	0.0000	0.0000
Right caudate	(13,10,10)	7.73	0.0000	0.0000
Right accumbens	(9,12,−7)	7.62	0.0000	0.0000
Left frontal orbital cortex	(−30,24,−17)	6.95	0.0000	0.0000
Right frontal orbital cortex	(29,23,−16)	5.99	0.0000	0.0001
Right amygdala	(23,−4,−18)	5.74	0.0000	0.0001
Anterior cingulate gyrus	(1,18,24)	5.63	0.0000	0.0001
Left putamen	(−25,0,0)	5.59	0.0000	0.0001
Right putamen	(25,2,0)	5.42	0.0000	0.0002
Right para cingulate gyrus	(7,37,23)	5.42	0.0000	0.0002
Left pallidum	(−19,−5,−1)	5.39	0.0000	0.0002
Left superior frontal gyrus	(−14,19,56)	5.39	0.0000	0.0002
Right inferior frontal gyrus	(52,28,8)	5.33	0.0000	0.0002
Left thalamus	(−10,−19,6)	5.27	0.0000	0.0002
Subcallosal cortex	(−0,21,−15)	5.21	0.0000	0.0002
Left para cingulate gyrus	(−6,37,21)	4.71	0.0001	0.0006
Left temporal pole	(−40,11,−30)	4.34	0.0002	0.0016
Left amygdala	(−23,−5,−18)	4.12	0.0004	0.0027
Right accumbens	(9,12,−7)			
Left accumbens	(−9,11,−7)	7.62	0.0000	0.0000
Right caudate	(13,10,10)	6.87	0.0000	0.0000
Left caudate	(−13,9,10)	6.75	0.0000	0.0000
Right amygdala	(23,−4,−18)	6.40	0.0000	0.0000
Subcallosal cortex	(−0,21,−15)	5.43	0.0000	0.0003
Left amygdala	(−23,−5,−18)	5.11	0.0000	0.0006
Right temporal pole	(41,13,−30)	4.90	0.0000	0.0009
Right para cingulate gyrus	(7,37,23)	4.84	0.0001	0.0009
Right frontal orbital cortex	(29,23,−16)	4.74	0.0001	0.0011
Anterior cingulate gyrus	(1,18,24)	4.67	0.0001	0.0012
Right inferior temporal gyrus	(46,−2,−41)	4.56	0.0001	0.0014
Right hippocampus	(26,−21,−14)	4.49	0.0001	0.0015
Left inferior temporal gyrus	(−48,−5,−39)	4.16	0.0003	0.0033
Right thalamus	(11,−18,7)	4.08	0.0004	0.0038
Left frontal orbital cortex	(−30,24,−17)	4.01	0.0005	0.0042
Right putamen	(25,2,0)	3.92	0.0006	0.0050

*FDR, false discovery rate; ROI, region of interest; RW, rested wakefulness. p < 0.005, FDR set-wise corrected for all comparisons*.

**Table 3:** ROI-to-ROI functional connectivity statistics of the NAc following TSD.

**Target region**	**MNI center**	***t***	**Uncorrected**	**FDR-corrected**
			***p*-value**	***p*-value**
Left accumbens	(−9,11,−7)			
Left caudate	(−13,9,10)	11.08	0.0000	0.0000
Right accumbens	(9,12,−7)	8.18	0.0000	0.0000
Subcallosal cortex	(−0,21,−15)	7.61	0.0000	0.0000
Right caudate	(13,10,10)	7.53	0.0000	0.0000
Right amygdala	(23,−4,−18)	6.18	0.0000	0.0000
Right putamen	(25,2,0)	5.86	0.0000	0.0001
Left putamen	(−25,0,0)	5.46	0.0000	0.0002
Left amygdala	(−23,−5,−18)	4.99	0.0000	0.0006
Right inferior temporal gyrus	(46,−2,−41)	4.71	0.0001	0.0011
Left pallidum	(−19,−5,−1)	4.70	0.0001	0.0011
Right para hippocampal gyrus	(22,−8,−30)	4.42	0.0002	0.002
Left superior frontal gyrus	(−14,19,56)	4.38	0.0002	0.002
Right pallidum	(20,−4,−1)	4.35	0.0002	0.002
Frontal medial cortex	(0,43,−19)	4.25	0.0003	0.0025
Right inferior frontal gyrus	(52,28,8)	−4.04	0.0004	0.0039
Right temporal fusiform cortex	(−32,−4,−42)	3.97	0.0005	0.0044
Left temporal fusiform cortex	(31,−3,−42)	3.92	0.0006	0.0047
Right accumbens	(9,12,−7)			
Left accumbens	(−9,11,−7)	8.18	0.0000	0.0000
Left caudate	(−13,9,10)	7.67	0.0000	0.0000
Right caudate	(13,10,10)	5.93	0.0000	0.0001
Subcallosal cortex	(−0,21,−15)	5.53	0.0000	0.0003

*FDR, false discovery rate; ROI, region of interest; RW, rested wakefulness. p < 0.005, FDR set-wise corrected for all comparisons*.

### Altered NAc Connectivity After TSD

**Figure 3:**
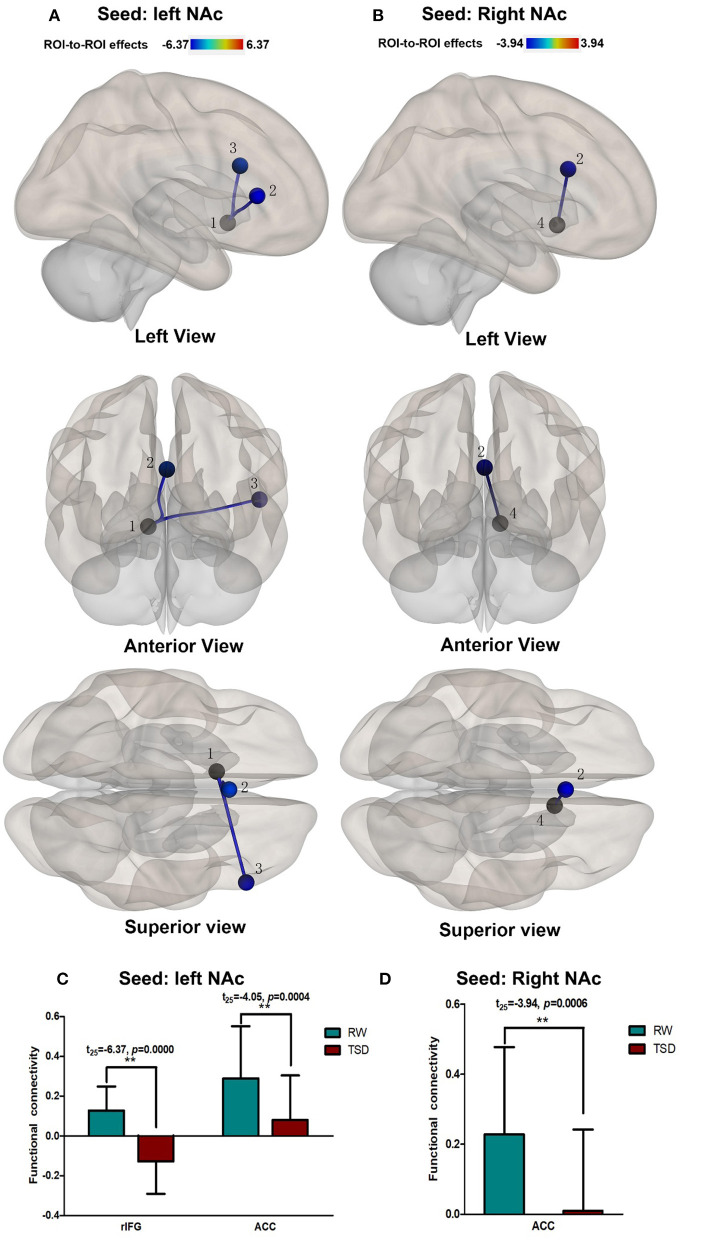
Alerted ROI-to-ROI functional connectivity of NAc in RW vs. TSD scans. **(A,C)** Left NAc. **(B,D)** Right NAc. **(A,B)** The position of functional connectivity from left, anterior, and superior views; **p < 0.001, uncorrected for all comparisons. **(C,D)** Functional connectivity effect size. ROI, region of interest; TSD, total sleep deprivation; RW, rested wakefulness; ^1^left NAc; ^3^rIFG; ^2^ACC; ^4^right NAc; rIFG, right inferior frontal gyrus; ACC, anterior cingulate cortex; NAc, nucleus accumbens.

Compared with RW, participants showed reduced functional connectivity between the left NAc and right IFG (rIFG) (*t* = −6.37, p_FDR−corrected_ < 0.05) and ACC (*t* = −4.05, p_FDR−corrected_ < 0.05) after TSD. Furthermore, reduced functional connectivity between the right NAc and ACC (t = −3.94, p_Uncorrected_ < 0.001) was found after TSD (Table 4, Figure 3).

**Table 4:** ROI-to-ROI functional connectivity statistics the NAc: comparisons between RW and TSD.

**Target region**	**MNI center**	***t***	**Uncorrected**	**FDR-corrected**
			***p*-value**	***p*-value**
Left accumbens	(−9,11,−7)			
Right inferior frontal gyrus	(52,28,8)	−6.37	0.0000	0.0002
Anterior cingulate gyrus	(1,18,24)	−4.05	0.0004	0.0287
Right accumbens	(9,12,−7)			
Anterior cingulate gyrus	(1,18,24)	−3.94	0.0006	0.0682

*ROI, region of interest; FDR, false discovery rate; RW, rested wakefulness; TSD, total sleep deprivation. p < 0.001, uncorrected for all comparisons*.

### Correlation Between Connectivity Strength and Distress

**Figure 4:**
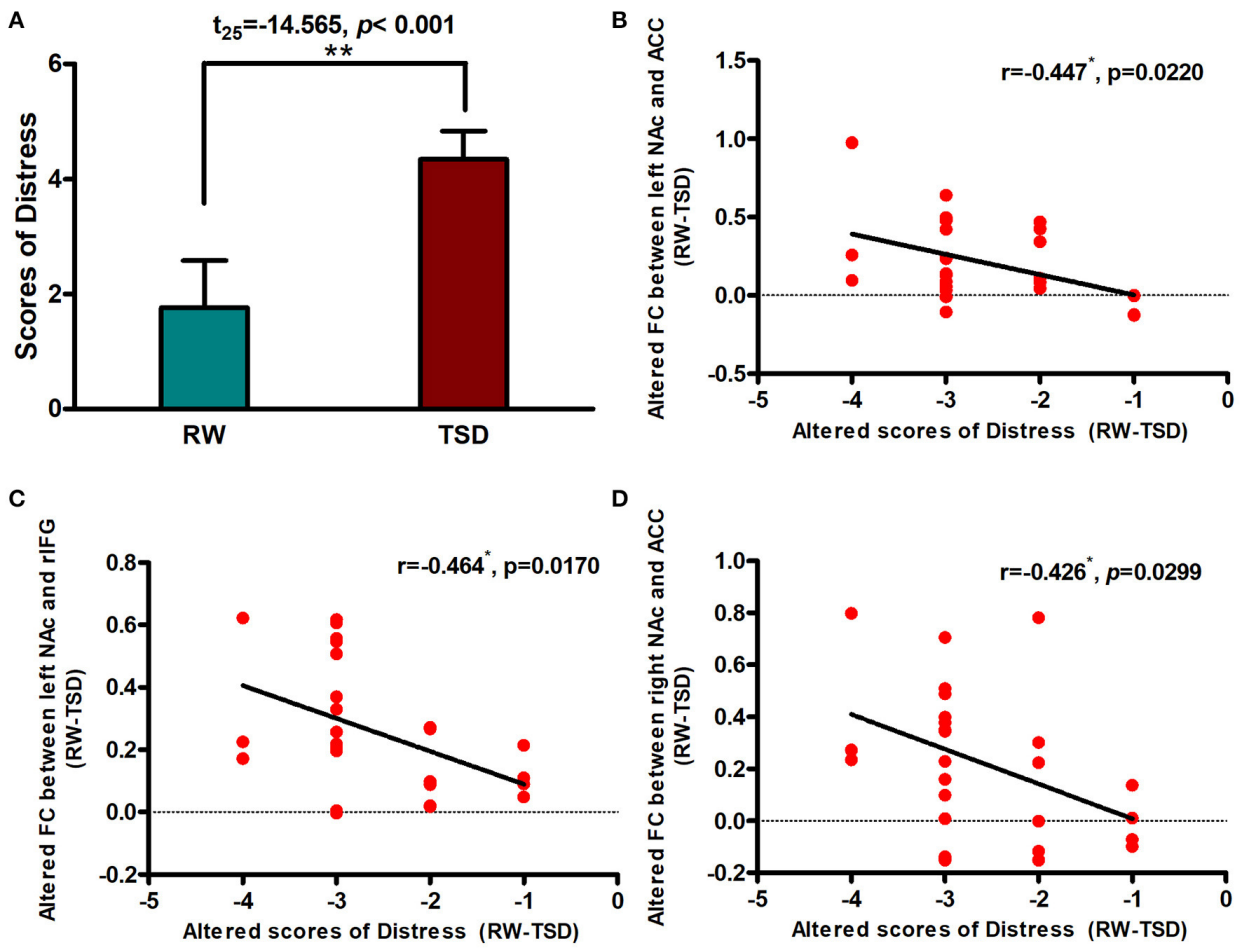
Alerted functional connectivity was negatively correlated with Distress. **(A)** Distress score in RW vs. TSD scans. Alerted score of Distress was negatively correlated with the alerted functional connectivity between the **(B)** left NAc and ACC, **(C)** left NAc and rIFG, and **(D)** right NAc and ACC. ROI, region of interest; TSD, total sleep deprivation; RW, rested wakefulness; rIFG, right inferior frontal gyrus; ACC, anterior cingulate cortex; NAc, nucleus accumbens. *p < 0,05, **p < 0.01.

After SD, distress scores increased significantly after TSD when compared with RW (*t* = −14.565, *p* < 0.001; Figure 4A). We performed Pearson correlation analysis between the alterations in brain functional connectivity and the degree of distress score increase. We found that there was a significant negative correlation between them (r left NAc-ACC = −0.447, p_FDR−corrected_ = 0.0333; r right NAc-ACC = −0.426, p_FDR−corrected_ = 0.0299; Figures 4B,D). The correlation was also found between left NAc and rIFG, but the difference was only marginally significant after FDR correction (left NAc-rIFG = −0.464, p_FDR−corrected_ = 0.051; Figure 4C).

## Discussions

We found that there was a decline in functional connectivity between the NAc and ACC and rIFG following TSD. Furthermore, the functional connectivity between these areas was negatively correlated to negative emotional state. Based on our results, we can deduce that the functional connectivity alteration in the reward network significantly impacted the TSD-induced enhancement of negative emotion. To the best of our knowledge, this is one of the few studies to use fcMRI to investigate functional connectivity changes in the reward network associated with SD. This study enriches experimental evidence in brain functional imaging for the negative emotional processing after TSD.

Consistent with the results of some studies, we found that SD could increase negative emotions (28). But this contradicted the therapeutic effect of SD on depression (29). We think one of the reasons may be that all the subjects, used in the study of SD in the treatment of depression, were depressed patients. Their emotional baseline was different from that of normal people and negative emotions was stronger than normal people. This may be one of the reasons why sleep deprivation has different effects on the mood of patients with depressed and normal people.

The NAc plays an important role in reward processing in the brain (30). It generates information on the value of stimuli, which is transferred to the cingulate gyrus and orbital frontal gyrus for value judgment (31, 32). This process has been linked to drug addiction and relapse. As the length of SD is extended, the need for sleep can be aroused. Sleep is a protective factor that is regulated by the reward system in the brain (22). The ACC is a key point in reward network because it controls and analyzes information from the NAc and ventral tegmented area. The functional connectivity between NAc and ACC found in this study may be a response to sleep requirements. The present findings support the hypothesis that the reward processing network is less able to communicate information after SD than during wakefulness. This may lead to the aggravation of a negative evaluation of the individual's inability to sleep; therefore, affecting the individual's behavioral response during SD.

Additionally, we found decreased functional connectivity between the NAc and rIFG following TSD. The NAc is regulated by the ACC and IFG (33). The limbic system provides value judgments on reward and punishment information; therefore, decreased rIFG functional connectivity may impact on the processing of emotional value after SD. Following information processing in the NAc, judgment values are passed to the medial prefrontal cortex, where executive decision-making occurs with the IFG (21, 34). This information is controlled by the IFG to guide individual behavior (35). Many studies show that rIFG is related to executive control and is part of the executive control network (36). The reward network system is regulated by the executive control system; therefore, our results suggest that there is a reduction in functional synchronization between important nodes of the executive control and reward networks. This may also impact the coordination of processing and guiding reward information. Research has shown that a loss of control in the subcortical nuclei of the reward network leads to unconquerable addictive behavior (32, 37). It is generally accepted that the reward system is regulated by the IFG, which is an important brain area of acquired development, plays a key role in human decision-making and behavior response inhibition, and regulates human behavior through feedback at all times. When this regulation is imbalanced, it can lead to a series of negative reactions, such as withdrawal reaction, relapse, and the occurrence of negative emotions. Decreased functional connectivity between the rIFG and NAc may be a manifestation of a decreased ability to process and control negative reward information.

Few studies have focused on the reward network and emotional state. Most studies have suggested that negative reflections can induce a deterioration in the emotional state (38). To verify the relationship between functional connectivity changes and behavioral responses, we further analyzed changes in the functional connectivity of the brain after SD and negative emotional states. Our results showed that functional connectivity between the NAc and ACC, and the NAc and rIFG were correlated with increased negative emotions following SD. Following SD, individuals experience increased negative emotions and stronger negative reactions to images (11). This study found that the increase in negative emotions, especially distress, after TSD was negatively correlated with a decrease in functional connectivity between the NAc, ACC, and rIFG. Although after multiple comparison correction, the correlation between alterations in functional connectivity of rIFG and the degree of distress score increase was only marginally significant. This offers valuable information regarding negative emotional processing after TSD and reflects a possible role for the reward system in the negative emotional processing. Our results support this increase in negative emotions following SD and offer a novel association with reward processing. We found that emotional instability was correlated with frontal cortex network instability, indicating that SD affected negative value judgments, leading to an enhanced negative emotion state.

The reward reaction includes negative punishment and positive reinforcement. Gujar et al. have shown a relationship between positive emotion responses after SD and the reward system. Furthermore, they found that SD is associated with enhanced reactivity toward negative stimuli, but imposes a bidirectional response to affective imbalance, which is associated with amplified reward-relevant reactivity toward pleasure-evoking stimuli (20). We found no significant correlation between positive POMS scores and changes in functional connectivity. This may be because the emotional response of individuals following TSD is primarily negative when there is no reward stimulus.

There are some limitations in our study. First, we did not explore objective ratings of emotional stimuli, such as sleep need; therefor this study is preliminary. We did not use the value judgment scale with the POMS scale; therefore, we can assess correlations between changes in emotional state and functional connectivity between the NAc and ACC, but we cannot directly assess processing the value of emotional states. Secondly, there are similarities and differences in the brain regions of the reward and emotional networks; therefore, changes in functional connectivity between the two networks need to be further explored. An emotion task should be included in future studies to investigate changes in brain activity within the reward networks after SD. Future studies should assess the interactions between brain networks. Thirdly, there is a lack of female participants in our study, which limits the extrapolation of the research results. This will provide further understanding of the mechanisms underlying changes in emotional states after SD.

Taken together, our data have identified a possible mechanism for emotional instability following sleep restriction. We found that SD decreased functional connectivity within the reward network. This decline in functional connectivity may be associated with enhanced negative emotional experiences after total SD, as we found that ‘distress’ was significantly correlated with changes in reward network connectivity. Further studies are required to assess the effect of lengthy sleep restriction on the processing of reward values, and the relationship between changes in the reward network and emotional instability.

## Data Availability Statement

The datasets generated for this study are available on request to the corresponding author.

## Ethics Statement

The studies involving human participants were reviewed and approved by Ethics Committee of The General Hospital of PLA (Beijing, China). The patients/participants provided their written informed consent to participate in this study.

## Author Contributions

YZ contributed substantially to acquisition, analysis, and interpretation of the data, and drafted the article. CD revised the manuscript, uploaded it, and would respond to the possible revision comments in the future. JP helped to analysis and interpretation of the data. YY, YS, and YH are the guarantors of this study and had complete access to all data in the study. They contributed substantially to conception and design as well as the interpretation of data. All authors listed have made a substantial, direct and intellectual contribution to the work, and approved it for publication.

## Conflict of Interest

The authors declare that the research was conducted in the absence of any commercial or financial relationships that could be construed as a potential conflict of interest.
